# Retraction: IL-6 and IL-10 Anti-Inflammatory Activity Links Exercise to Hypothalamic Insulin and Leptin Sensitivity through IKKβ and ER Stress Inhibition

**DOI:** 10.1371/journal.pbio.3002079

**Published:** 2023-04-04

**Authors:** The PLOS Biology Editors

Following the publication of this article [1], concerns were raised regarding results presented in Figs 2, 3, 4, 6, 7, and 8. Specifically,

- The following western blot panels appear similar:
  ○ Lanes 2–5 of the Fig 7F IB:IKKα panel and lanes 1–4 of the Fig 7J IB: STAT3 panel when flipped vertically.
  ○ Lanes 1–3 of the Fig 2A IB:α-tubulin panel of this study [1] and lanes 1–3 of the Fig 1B IB:α-tubulin panel of [2, retracted in 3] when flipped horizontally.
  ○ Lanes 2–4 of the Fig 2F IB: pJak2^Tyr1007/8^ panel of this study [1] and the Fig 2E pAMPK^Thr172^ panel of [4].
  ○ Lanes 1–4 of the Fig 3H IB:α-tubulin panel of this study [1] and lanes 3–6 of the Fig 2D IB:pIRβ panel of [2, retracted in 3] when flipped vertically.
  ○ Lanes 2–5 of the Fig 6B IB:α-tubulin panel of this study [1] and the Fig 5A IB:βActin panel of [5, retracted in 6].
  ○ The Fig 8G IB: α-tubulin panel of this study [1] and the Fig 2C IP:JAK2 IB:JAK2 panel of [2, retracted in 3].
  ○ The Fig 8H IB: α-tubulin panel of this study [1] and the Fig 5B IB: α-tubulin panel of [2, retracted in 3].
- The following Fig 4E confocal microscopy panels appear to partially overlap:
  ○ The left half of the top DAPI panel, and the right half of the middle DAPI panel.
  ○ The left half of the top IL-6R panel, and the right half of the middle IL-6R panel panel.
  ○ The left half of the IKKβ panel, and the right half of the PERK panel.
  ○ The left half of the top Merge panel, and the right half of the middle Merge panel.

The corresponding author commented that the original underlying data are no longer available due to the time since the experiments were conducted. In the absence of the underlying data, the issues with this article cannot be resolved.

In light of the concerns affecting multiple figure panels that question the integrity of these data, the *PLOS Biology* Editors retract this article.

The Fig 2F IB: pJak2Tyr1007/8 panel reports material from [4], published in 2007 by the Endocrine Society, which is not offered under a CC-BY license and is therefore excluded from this article’s [1] license. At the time of retraction, the article [1] was republished to note this exclusion in the Fig 2 legend and the article’s copyright statement.

ERR, GZR, DG, AGO, MJAS, and JBCC agreed with the retraction. ERR, GZR, DG, MJAS, and AGO stand by the article’s findings and apologize for the issues with the article. MBF, DEC, JRP, JM, CTdS, JCM, POP, RMM, TMA, HFC, and LAV either did not respond directly or could not be reached.
